# Unravelling the dilemma of self-medication in Egypt: a cross-sectional survey on knowledge, attitude, and practice of the general Egyptian population

**DOI:** 10.1186/s12889-024-17913-3

**Published:** 2024-03-01

**Authors:** Hossam Tharwat Ali, Mostafa Barakat, Ahmed Reda Abdelhalim, Ibrahim Noureddin Al-Kurd, Muhammad Kamal-Eldeen Muhammad, Mohamed Mostafa Sharkawy, Mohamed Elbahnasawy, Samar Ahmed Amer, Self-Medication Collaborative Group, Muhammad Masoud, Mahmoud Ahmed Rabea, Abdelrahman Aboelala, Eman Ayman Nada, Sara Abdelhameed Khalil, Amal M. Sharaf, Bassant Ashraf Ibrahim, Hanin Abdelhamied Rabea, Nourhan Omar, Yosra Hussein AboEl-Azm, Sohieb Hedawy, Abdelrahman Hendawy

**Affiliations:** 1 Qena Faculty of Medicine, South Valley University, Qena, 83621 Egypt; 2 Faculty of Medicine, Alexandria University, Alexandria, Egypt; 3 Faculty of Medicine, Benha University, Benha, Egypt; 4 Emergency Medicine and Traumatology Department, Faculty of Medicine, Tanta University, Tanta, Egypt; 5 Department of Public Health and Community Medicine, Family Medicine, Membership at Royal College of General Practice [INT], Ministry Of Health (MOH) Saudi Arabia, Zagazig University, EgyptLondon, UK

**Keywords:** Egypt, Self-medication, Knowledge, Practice, Attitude

## Abstract

**Background:**

Self-medication (SM) is a rising public health issue, especially in developing countries. It can be associated with various problems such as the delayed seeking of medical advice, drug interactions, and serious events such as antimicrobial drug resistance. We aimed to evaluate the Egyptian general population’s knowledge, attitudes, and practices of SM.

**Methods:**

We employed a cross-sectional design between February 7th and March 8th, 2023 using a self-administered questionnaire available in Arabic. The questionnaire was developed based on previous studies and included four domains: sociodemographic data, knowledge, attitude, and practice of SM. We utilized both online (Google Forms) and paper surveys, utilizing convenience and snowball sampling methods. Data were analyzed using R Statistical Software (v4.1.3; R Core Team 2022).

**Results:**

1630 Egyptian individuals (838 females and 792 males) from the seven provinces were enrolled, with a median age of 25 years (IQR: 22–40). Around 55.97% and 48.28% of the participants had good knowledge and favorable attitudes regarding SM respectively, while 62.8% had practiced SM in the previous three months. The most frequently used medications were painkillers (60.74%) followed by antibiotics (32.13%) and antipyretics (28.61%). The pharmacist’s recommendation was the source of SM for 53.61% while 31.53% used old medications at home. Most participants (59.08%) practiced SM because they thought they had simple or minor symptoms. The multivariate regression analysis revealed that females had significantly higher knowledge of SM than males (aOR: 2.10; 95%CI: 1.64—2.71; *p*-value < 0.001), with no significant differences in practice (aOR: 1.24; 95%CI: 0.99 – 1.56; *p*-value = 0.065). Individuals working or studying in the medical field were significantly more knowledgeable about SM (aOR: 4.30; 95%CI: 3.27–5.69; *p*-value < 0.001) and more likely to practice SM (aOR: 1.65; 95%CI: 1.26–2.17; *p*-value < 0.001). The odds of SM decreased with favorable attitudes (aOR: 0.44; 95%CI: 0.36–0.55; *p*-value < 0.001) while surprisingly, knowledge level was not significantly contributing to SM practice (aOR: 1.15; 95%CI: 0.90–1.48; *p*-value = 0.268).

**Conclusions:**

SM is prevalent in Egypt, highlighting the importance of raising awareness and encouraging physician consultation as a priority. Governments, healthcare organizations, and educational institutions need to collaborate to provide the necessary support and resources.

**Supplementary Information:**

The online version contains supplementary material available at 10.1186/s12889-024-17913-3.

## Introduction

Self-medication (SM) is defined by the World Health Organization (WHO) as an element of health care in which a person is medicated to treat self-diagnosed disorders or illnesses. Individuals may consume medications without the guidance of doctors which can be of many sources such as reusing old prescriptions or sharing drugs with friends or relatives [1, 2]. This consumption may be of over-the-counter drugs (OTCs) and/or prescription-only medications (POMs). OTCs are usually used in minor illnesses and are cheaper so they are more widely self-used especially in economic crises [3].

SM is a global health issue that is fluctuating and expanding among various populations worldwide. As expected, it is more prevalent in developing countries [4]. It ranges from 11.20% to 93.70% depending on the population and country [5]. In China, a survey found that about 38.00% of those who got ill did not seek medical advice, and 72.00% of them preferred SM [6]. A study in Britain surveyed the prevalence of SM and found that 93.00% of patients experienced pain within one month, and of these 75.00% chose the easier way of SM [6]. Even in the USA, a study observed that about 72.00% of people who suffered from symptoms like cough, headache and cold chose SM, primarily [6]. In Saudi Arabia, a study revealed that about 81.4% of the general population have practiced SM at least one time in their life [4]. During the coronavirus disease 2019 (COVID-19) pandemic, SM was prevalent in the Arab region with around two-thirds of the population reporting practicing SM during the pandemic. Noteworthy, Egypt had the highest prevalence (72.10%) while Palestine had the lowest prevalence (40.40%) [7].

Adherence to the proper practical guidelines and restrictions for the use of SM saves time and money and lessens the load on medical services [4]. However, improper use in the form of unnecessary conditions, improper doses, and/or duration of intake is more common. Such practices may lead to irrational drug use, delayed seeking of medical advice, drug interactions, and increased risk of adverse drug events, or serious events such as antimicrobial drug resistance [4, 8].

One's use pattern of SM may depend on or be influenced by several variables, e.g., age, sex, socioeconomic status, level of education, place of residence, field of study, lifestyle traits (smoking and drinking habits), health-related traits (chronic disease conditions or the presence of mild health problems), and cultural factors [9–11]. The variety of pharmaceuticals worldwide eases accessibility [12], and unregulated access especially in developing countries allows even prescription-only medications to be self-medicated [9]. Lack of low-cost consultation and trust in medical doctors may be contributing factors to this phenomenon in certain populations [4].

Despite the widespread acknowledgment of SM worldwide and high rates of medication misuse among the Egyptians [13–17], to the authors' knowledge, no study investigated that problem on the extensive level of the Egyptian general population. All previous studies included fewer participants from only one or two provinces or universities per study. The largest study was done on adults attending pharmacies in Alexandria, showing a prevalence of 81.10% for SM [17]. Others were performed on university students of one university or city (Mansoura, Ain Shams, Suez Canal, and Cairo universities) per each study, revealing a prevalence of SM ranging from 38.20% to 91.10% among Egyptian university students [4, 9, 18, 19]. Therefore, this study endeavors to bridge the existing knowledge gap pertaining to SM among the Egyptian general population. Our objectives are to evaluate their knowledge of and attitudes toward the subject matter and to ascertain whether any notable contributing factors. Furthermore, we intend to identify reasons for and patterns of SM among the general population. Lastly, through the dissemination of our findings, we aim to promote consciousness about the significance of SM.

## Methods

### Study design and population

We conducted a cross-sectional study among the Egyptian general population in all provinces or regions including Greater Cairo, Alexandria, Suez Canal, Delta, Northern Upper Egypt, Southern Upper Egypt, and Asyut regions. The study was done between February 7th and March 8th, 2023, using online and/or paper surveys. Egyptians of any gender, aged 18 years old or above, and able to respond to the questionnaire in the Arabic language were invited to participate in the study.

### Sampling and sample size calculation

Convenience and snowball sampling methods were used to recruit eligible study participants. The sample size was calculated using Epi Info statistical calculator 7.2.5. version, which is a trademark of the Centers for Disease Control and Prevention (CDC), with the following parameters: a confidence interval of 95.00%, an expected frequency of 50.00%, and an acceptable margin of error of 5.00%. The minimum sample size was 400 responses. The sample size was increased to 1630 to increase the power of the study and represent all regions and provinces.

### Study tool (questionnaire development)

The questionnaire was developed based on questions from previous studies [20, 7]. It was designed as a self-administered Google Form survey available in Arabic language. The questionnaire was divided into four domains: sociodemographic data, knowledge regarding SM, attitude toward SM, and practice of SM. The English version of the study questionnaire is available in Additional File 1.

Sociodemographic data included: age, sex, region (province) of residence, the original place of residence, educational level, employment, field of study or work, household income, medical insurance, and health status (allergies and chronic or congenital diseases).

The knowledge section included five questions about the definition of SM, the dangers of SM, and the timing of consulting a physician. The knowledge questions have been recoded as 2 for the correct answer and 0 for the incorrect one giving a total score from 0 to 10 for each participant. The average score of the knowledge section was used as the cut-off limit as adopted in many knowledge, attitude, and practice (KAP) studies [21–23]. Participants with a score that is equal to or above the mean score were considered to have a good level of knowledge while others with less than the mean score were considered to have a poor level of knowledge regarding SM.

Attitudes towards the SM section included five Likert scale questions on SM as a part of self-care, recommending SM to others, and the ability of the general population to recognize, diagnose, and treat diseases in addition to using medications properly. The questions of the attitude section were recoded as follows; completely disagree = 5, disagree = 4, neutral = 3, agree = 2, and completely agree = 1, giving a total score from 5 to 25 for each participant. The mean score of the attitude section was deemed the cut-off point and participants with a score that is equal to or above the mean score were considered to have a favorable attitude while those with less than the mean score were considered to have an unfavorable attitude.

The practice of SM section included nine questions on SM practice in the last three months and if practiced, frequency, if those medications helped, if there were any side effects of those medications, how the COVID-19 pandemic affected participants’ habit of SM, types, sources, and information of those medications as well as reasons for practicing SM.

### Pilot study and validation

To validate the content of the survey, three experts in public health and medicine were invited to fill in the survey and assess the clarity, comprehension, and relevance of each question to the measured outcome (knowledge, attitude, or practice). We adjusted the questionnaire to ensure both relevance and feasibility among our population according to the experts’ comments. Afterward, a pilot study was conducted over three days and included 162 responses. Comments of the collaborators and participants on the questions’ clarity, comprehension, and wording were retrieved and considered before data collection provided that they do not affect the questions’ relevance. Additionally, the reliability and internal consistency of the survey were assessed using Cronbach’s alpha which was 0.63 for the knowledge section and 0.72 for the attitude section which were considered acceptable for internal consistency [24].

### Data collection

An online link to the Google form was distributed on the different social media platforms with the help of the study collaborators. The link recorded the data anonymously and did not record any contact or personal information. Individuals who may not have access to the internet or the link were approached through public places such as roads and libraries in addition to family gatherings and invited to participate and fill in the paper questionnaire. Paper questionnaires were then entered by the study collaborators.

At the beginning of the survey, the individual had the option to consent or decline to participate in the study. Afterward, we set a confirmatory question to ensure that the individual has not filled in the questionnaire for the same study before to prevent duplicate data. Participants with incomplete responses were all excluded to prevent information bias.

### Ethical considerations

The study was conducted according to the principles expressed in the Declaration of Helsinki. Participation in this survey was voluntary. Informed consent was obtained from all subjects. Participants’ anonymity and confidentiality were ensured throughout the study including data collection and analysis. Ethical approval was obtained from the institutional review board committee (IRB) at Tanta University, Faculty of Medicine (approval code: 36264PR32/1/23).

### Data analysis

The data were organized in a Microsoft Excel sheet and then imported and analyzed using R Statistical Software (v4.1.3; R Core Team 2022). For baseline demographic characteristics, frequencies, and percentages were used to describe the categorical variables. Shapiro test revealed age was not normally distributed so was described as median and interquartile range.

Univariate and multivariate regression analyses were performed including all demographic characteristics as independent variables for the knowledge regression model whereas for the attitude regression model, we included the demographic variables as well as knowledge levels as independent variables. The practice model included demographic characteristics, knowledge level, and attitude levels as independent variables. The results were reported as odds ratio (OR) and 95.00% confidence intervals (CI). A p-value of ≤ 0.05 was considered significant.

## Results

In total, 1817 individuals were invited to fill in the survey: 1263 through the online survey and 554 through the paper survey. 14 participants refused to participate in the study and only 1803 completed the survey. The forms of 173 participants were excluded due to inconsistencies and the final analysis included responses of 1630 participants.

### Demographic characteristics of the participants

Of the included 1630 participants, 838 (51.41%) were females. The median age of our participants was 25 and the interquartile range of 22 to 40 years old. Of the seven provinces of Egypt, Southern Upper Egypt had the highest rate of responses (26.13%) followed by the Grater Cairo region (24.17%) while the Asyut region had the lowest response rate (3.74%). More than half of our participants were residents of urban areas (61.53%), single (58.40%), had a university education or above (63.37%), were working or studying in a non-medical field (57.79%), and did not have health insurance (56.38%), history of drug allergy (63.00%), or chronic or congenital diseases (69.69%). Of those with chronic or congenital diseases, cardiovascular diseases such as hypertension were the most prevalent (46.77%) followed by musculoskeletal disorders (31.78%) and endocrinal disorders such as diabetes mellitus (21.86%). The details of the demographic characteristics of the participants are shown in Tables 1 and 2.

**Table 1:** Demographic characteristics of the participants

Variable	Levels	Frequency (%) (*N* = 1630)
**Age** (years) Median (IQR)		25 (22 to 40)
**Gender**	Female	838 (51.41)
	Male	792 (48.59)
**Region or province**	Southern Upper Egypt	426 (26.13)
	Greater Cairo Region	394 (24.17)
	Delta Region	287 (17.61)
	Alexandria Region	211 (12.94)
	Suez Canal Region	126 (7.73)
	Northern Upper Egypt Region	125 (7.67)
	Asyut Region	61 (3.74)
**Residence**	Urban	1003 (61.53)
	Rural	627 (38.47)
**Marital status**	Single	952 (58.40)
	Married	615 (37.73)
	Widow/Widower	35 (2.15)
	Divorced	28 (1.72)
**Highest educational degree**	Higher (university) education or above	1033 (63.37)
	High or secondary school	517 (31.72)
	Primary or elementary education	80 (4.91)
**Employment**	Not working	802 (49.20)
	Working in governmental work	326 (20.00)
	Working in private sector	265 (16.26)
	Free work such as freelancer, dayworker	200 (12.27)
	Retired	37 (2.27)
**Field of study or work**	Non-medical	942 (57.79)
	Medical	688 (42.21)
**Does the household income suffice the basic requirements of the family?**	It barely suffices	914 (56.07)
	It is not sufficient	374 (22.94)
	It is more than sufficient	342 (20.98)
**Do you have health insurance?**	No	919 (56.38)
	Yes	711 (43.62)
**Does the health insurance or monthly income support visiting a physician whenever you want?**	No	944 (57.91)
	Yes	686 (42.09)
**History of drug allergy**	No	1027 (63.01)
	Not sure	387 (23.74)
	Yes	216 (13.25)
**Medical history for chronic or congenital diseases**	No	1136 (69.69)
	Yes	494 (30.31)

Abbreviations *IQR* Interquartile range

**Table 2:** Chronic or congenital diseases in our patients

Disease	Frequency	Percentage (*N* = 494)
Cardiovascular diseases e.g. hypertension	231	46.77
Musculoskeletal disorders	157	31.78
Endocrinal disorders e.g. diabetes mellitus	108	21.86
Chronic headache	102	20.65
Respiratory conditions e.g. asthma	55	11.13
Blood diseases e.g. anemia	20	4.05
ENT disorders e.g. sinusitis	19	3.85
Kidney diseases	17	3.44
Liver diseases	16	3.24
GIT condition	14	2.83
Others ^a^	28	5.67

^a^Others include: Allergy, lupus, BPH, gout, PCOS, familial hypercholesterolemia, and endocrinal, neurological, dermatological, eye, ear, and psychiatric conditions

Abbreviations: ENT Ear, nose, and throat, *GIT* Gastrointestinal

### The participants’ knowledge of SM

Among the respondents, about 73.25% were aware of the definition of SM, whereas the percentages of those who did not know and were not sure about what SM means were very similar, 13.44 and 13.31 respectively. Around 60.37% considered taking medications of unknown sources or origin such as herbals not safe. Most of them (71.53%) knew that increasing the dose of medications without a physician’s consultation was not safe. Regarding consulting a physician in case any side effects occur, the majority (73.19%) of the participants considered that they should do so. About 66.63% believe that SM can not only hide serious symptoms and conditions but also lead to their exacerbation. The mean score of participants’ knowledge was 6.90 with a standard deviation of 2.73. Moreover, 54.97% of them were found to have good knowledge of SM, while 45.03% had poor knowledge. The participants’ responses to the knowledge questions are shown in Table 3.

**Table 3:** Knowledge of the participants regarding self-medication

Variable	Levels	Frequency (%) (*N* = 1630)
**Self-medication is taking medications without prescription or supervision of specialized physician**	No	219 (13.44)
	Not sure	217 (13.31)
	**Yes**	1194 (73.25)
**Taking medications of unknown sources or origin such as herbals is always safe**	**No**	984 (60.37)
	Not sure	355 (21.78)
	Yes	291 (17.85)
**Increasing the doses of medications without physician s supervision is always safe**	**No**	1166 (71.53)
	Not sure	60 (3.68)
	Yes	404 (24.79)
**In case side effects occur, we should consult a physician right away**	No	359 (22.02)
	Not sure	78 (4.79)
	**Yes**	1193 (73.19)
**Taking medication without physician’s supervision can hide serious symptoms or conditions and lead to its exaggeration**	No	159 (9.75)
	Not sure	385 (23.62)
	**Yes**	1086 (66.63)
**Total knowledge score** Mean (SD)		6.90 (2.73)
**Knowledge level**	Good (≥ mean)	896 (54.97)
	Poor (< mean)	734 (45.03)

The underlined answers are the correct answers

A good knowledge level is a score of ≥ mean of the total knowledge score

A poor knowledge level is a score of < mean of the total knowledge score

Abbreviations: *SD *Standard deviation

### The participants’ attitudes toward SM

The mean score of our participants’ attitudes was 19.11 with a standard deviation of 3.66. About 48.28% of participants had favorable attitudes towards SM, while 51.72% had unfavorable attitudes. The highest mean score for an item was 4.32 for (General population can prescribe medications properly without medical training) while the lowest mean score was 3.22 for (SM as a part of self-care). The attitudes of the participants are summarized in Table 4.

**Table 4:** Attitude of the participants toward self-medication

Item		Mean (SD)
**Self-medication is a part of self-care**		3.22 (1.13)
**General population can prescribe medications properly without medical training**		4.32 (0.85)
**Some people can properly recognize and diagnose diseases without consulting a physician**		3.92 (1.05)
**Some people can properly take medications on their own without consulting a physician**		3.63 (1.12)
**Self-medication is safe and I recommend it to my people**		4.01 (0.95)
**Attitude score**		19.11 (3.66)
**Attitude level** N (%)	Favorable (≥ mean)	787 (48.28)
	Unfavorable (< mean)	843 (51.72)

Abbreviations *SD* Standard deviation

### The participants’ practice of SM in the last three months

In this study, out of the 1024 (62.8%) individuals who practiced SM, 559 participants (54.6%) did so only less than three times during the last three months. More than half of them (58.3%) reported their practice of SM had not changed during the COVID-19 pandemic. Most of the participants knew the indications for use (72.46%) and the medication's name (70.61%) before they started to use the medication. Around 58.4% knew how to take or use the medication and 42.97% knew the proper dosage.

**Fig. 1:**
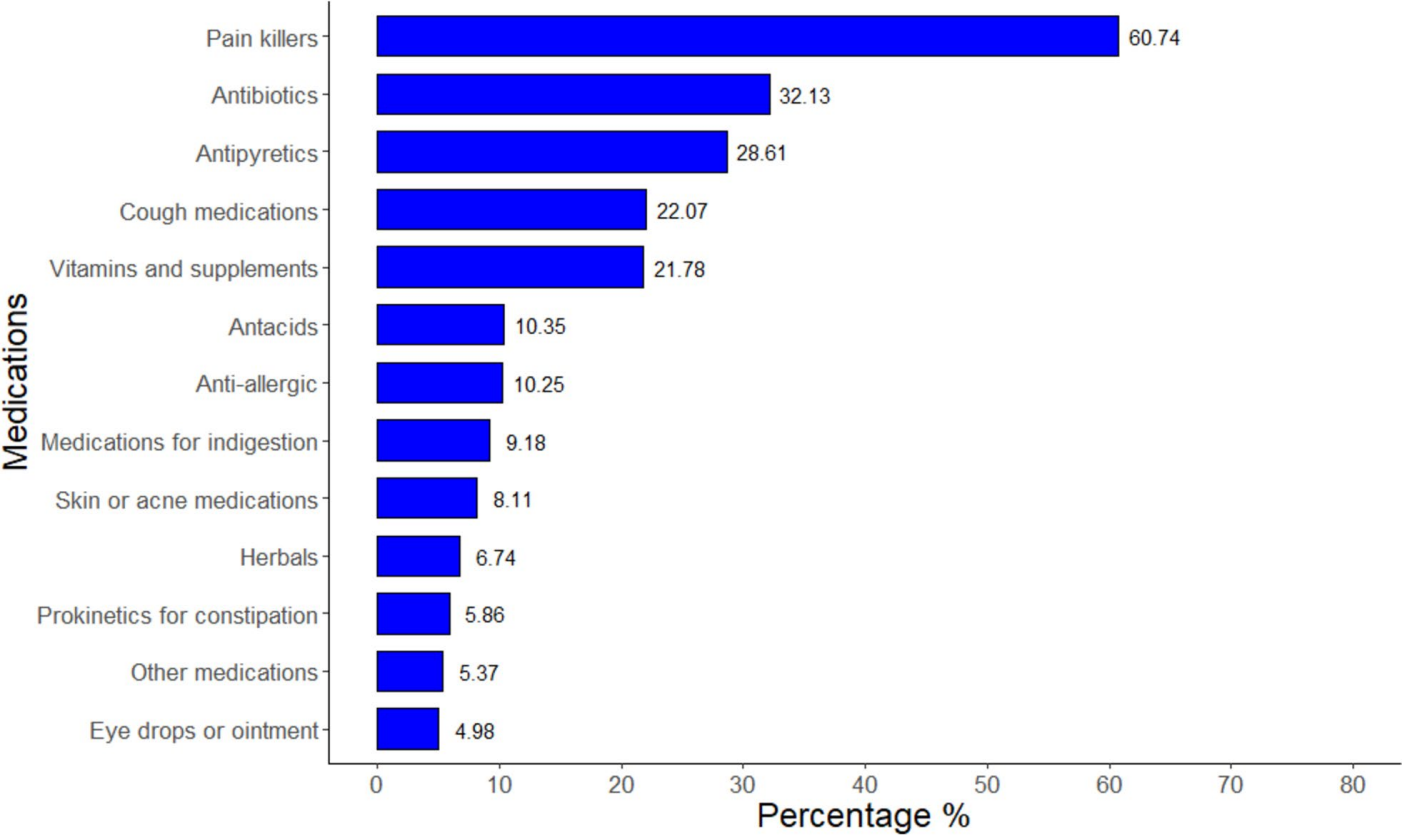
Medications that were used as self-medication among our participants

Painkillers were the most frequently self-used drugs in around 60.74% of those who practiced SM, followed by antibiotics (32.13%), antipyretics (28.61%), cough medications (22.07%), and vitamins and supplements (21.78%) (Fig. 1). The medications always work well for only 309 individuals (30.2%) while they sometimes work well for most of them (67.40%). The majority of those participants (82.10%) did not experience any side effects that necessitated medical advice.

**Fig. 2:**
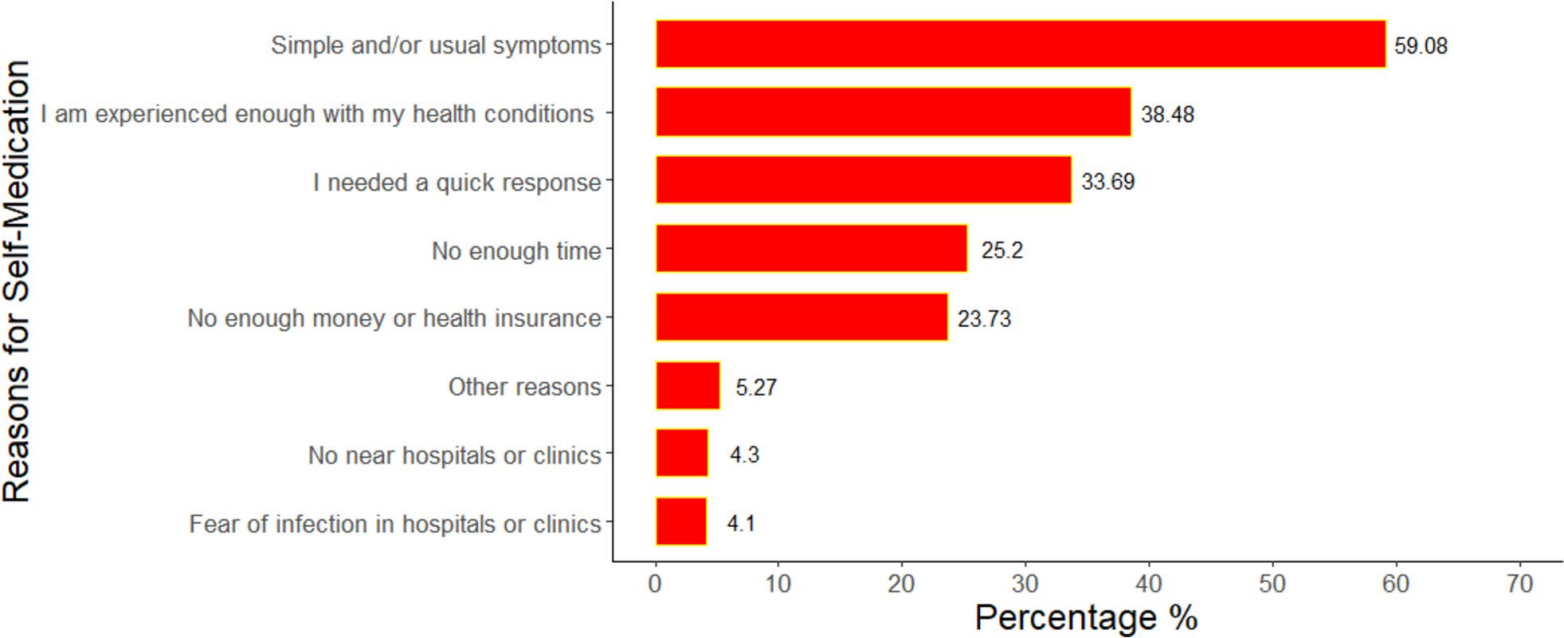
Reasons for self-medication among our participants

More than half of them (59.08%) took the medications because they thought they had usual or simple symptoms while 38.48% did so because they thought they were experienced enough and 33.69% did so because they needed a quick response. Nearly one-fourth did so due to lack of time and around 23.73% because they did not have enough money or health insurance (Fig. 2).

**Fig. 3:**
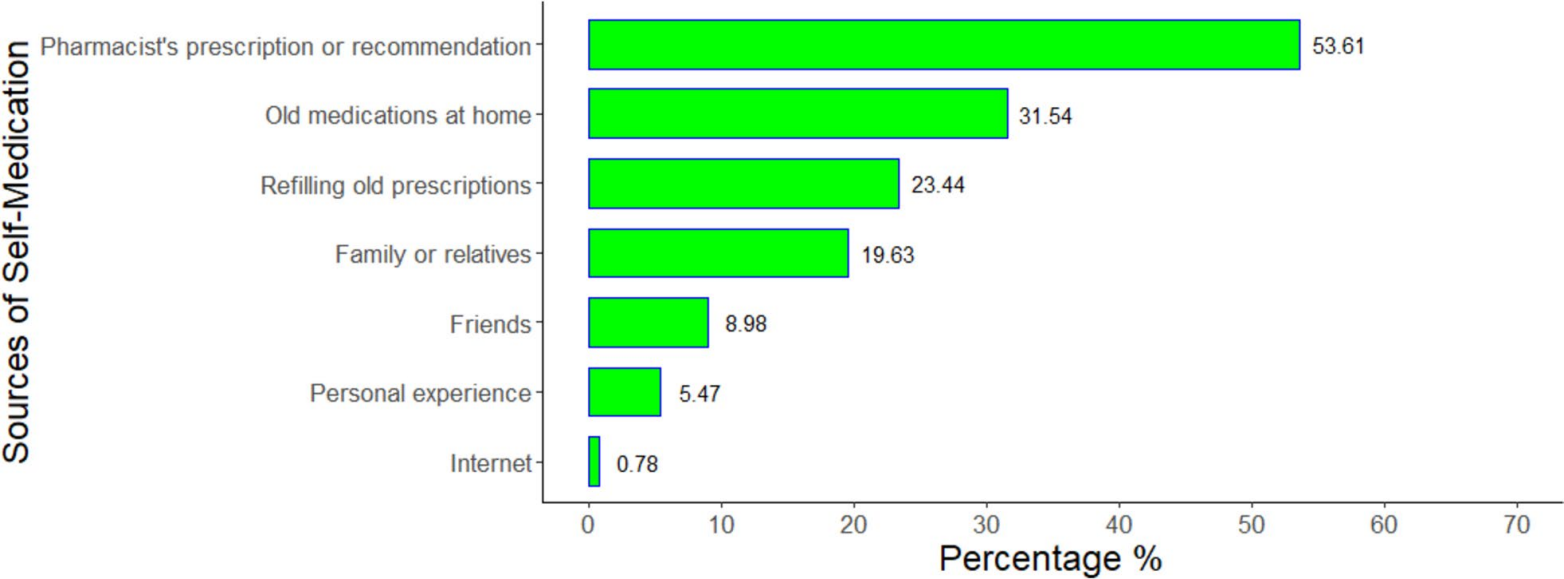
Sources of self-medication among our participants

Regarding the source of those medications, the pharmacist’s recommendation was the most common (53.61%) while around 31.54% and 23.44% of the participants who practiced SM used the old medications at home and refilled the previous prescriptions respectively. Nearly one-fifth of used medication from family or relatives while friends were the source for only 8.98% (Fig. 3). The details of the SM practices of our participants are shown in Table 5.

**Table 5:** Practice of self-medication among our participants

Variables	Levels	Frequency (%) (*N* = 1024)
**Have you taken medications on your own without the physician’s supervision or prescription during the last three months? (*N* = 1630)**	No	606 (37.20)
	Yes	1024 (62.80)
**How many times have you taken those medications in the last three months?**	Less than 3 times	559 (54.60)
	3—6 times	264 (25.80)
	More than 6 times	201 (19.60)
**Do these medications help you feel better?**	It gets worse	1 (0.10)
	No difference / I rarely feel better	24 (2.30)
	Sometimes I feel better	690 (67.40)
	I always feel better	309 (30.20)
**How did the COVID-19 pandemic affect your habit of self-medication?**	Decreased	73 (7.10)
	No change	597 (58.30)
	Increased	125 (12.20)
	I don't remember	229 (22.40)
**Did you have any side effects that necessitate physician consultation?**	No	841 (82.10)
	Not sure	87 (8.50)
	Yes	96 (9.40)
**Which of the following did you know before taking those medications? **^**a**^	Indications for use	742 (72.46)
	Medication name	723 (70.61)
	How to use or take the medication	598 (58.40)
	Proper dosage	440 (42.97)
	Possible side effects	276 (26.95)
	Duration of use or intake	274 (26.76)
	Contraindications	257 (25.10)
	Proper storage methods at home	211 (20.61)

^**a**^ Multiple response question

Abbreviations: COVID-19: Coronavirus disease 2019

### Univariate and multivariate analysis of the knowledge

In the multivariate analysis, females were found to have highly increased odds of having good knowledge (aOR: 2.1; CI: [1.64–2.71], *p*-value < 0.001). Married and divorced participants were found to have significantly decreased odds of having good knowledge in comparison to single participants (aOR:0.55; CI: [0.38–0.80], *p*-value = 0.002, and aOR:0.30; CI: [0.11–0.80], *p*-value = 0.018 respectively). University or higher education was shown to double the odds of having good knowledge in comparison to those with secondary or high education (aOR:2.12; CI: [1.60–2.83], *p*-value < 0.001). Participants with a more than sufficient household income were found to have increased odds of good knowledge (aOR:1.45; CI: [1.06–2.00], *p*-value = 0.021), while those with insufficient household income had fewer odds of good knowledge (aOR:0.54; CI: [0.40–0.73], *p*-value < 0.001) compared to those with a barely sufficient household income. The medical field of study or work was the factor with the highest significant odds of good knowledge (aOR:4.30; CI: [3.27–5.69], *p*-value < 0.001) compared to the non-medical field (Table 6).

**Table 6:** Univariate and multivariate logistic regression analysis of knowledge regarding self-medication among the study participants

**Dependent: knowledge level**		**Poor**	**Good**	**OR (95% CI, *p*-value) (univariable)**	**aOR (95% CI, *p*-value) (multivariable)**
**Age** (years)	Mean (SD)	36.2 (14.60)	28.1 (11.10)	0.95 (0.95–0.96, *p* < 0.001)	0.99 (0.98–1.01, *p* = 0.369)
**Gender**	Male	427 (53.90)	365 (46.10)	-	-
**Region or province**	Female	307 (36.60)	531 (63.40)	2.02 (1.66–2.47, *p* < 0.001)	2.10 (1.64–2.71, *p* < 0.001)
	Greater Cairo Region	219 (55.60)	175 (44.40)	-	-
	Alexandria Region	115 (54.50)	96 (45.50)	1.04 (0.75–1.46, *p* = 0.799)	1.22 (0.80–1.84, *p* = 0.357)
	Asyut Region	15 (24.60)	46 (75.40)	3.84 (2.12–7.32, *p* < 0.001)	3.04 (1.55–6.24, *p* = 0.002)
	Delta Region	113 (39.40)	174 (60.60)	1.93 (1.42–2.63, *p* < 0.001)	1.84 (1.26–2.70, *p* = 0.002)
	Northern Upper Egypt Region	55 (44.00)	70 (56.00)	1.59 (1.06–2.40, *p* = 0.024)	2.20 (1.37–3.55, *p* = 0.001)
	Southern Upper Egypt	161 (37.80)	265 (62.20)	2.06 (1.56–2.73, *p* < 0.001)	2.16 (1.54–3.03, *p* < 0.001)
	Suez Canal Region	56 (44.40)	70 (55.60)	1.56 (1.05–2.35, *p* = 0.030)	1.39 (0.85–2.27, *p* = 0.189)
**Residence**	Rural	283 (45.10)	344 (54.90)	-	-
	Urban	451 (45.00)	552 (55.00)	1.01 (0.82–1.23, *p* = 0.946)	0.94 (0.73–1.22, *p* = 0.662)
**Marital status**	Single	306 (32.10)	646 (67.90)	-	-
	Divorced	19 (67.90)	9 (32.10)	0.22 (0.10–0.49, *p* < 0.001)	0.30 (0.11–0.80, *p* = 0.018)
	Married	383 (62.30)	232 (37.70)	0.29 (0.23–0.35, *p* < 0.001)	0.55 (0.38–0.80, *p* = 0.002)
	Widow/Widower	26 (74.30)	9 (25.70)	0.16 (0.07–0.34, *p* < 0.001)	0.55 (0.20–1.48, *p* = 0.247)
**Highest educational degree**	High or secondary school	268 (51.8)	249 (48.20)	-	-
	Higher (university) education or above	396 (38.30)	637 (61.70)	1.73 (1.40–2.14, *p* < 0.001)	2.12 (1.60–2.83, *p* < 0.001)
	Primary or elementary education	70 (87.50)	10 (12.50)	0.15 (0.07–0.29, *p* < 0.001)	0.60 (0.27–1.26, *p* = 0.196)
**Employment**	Not working	307 (38.30)	495 (61.70)	-	-
	Free work such as freelancer, dayworker	141 (70.50)	59 (29.50)	0.26 (0.18–0.36, *p* < 0.001)	0.65 (0.43–0.99, *p* = 0.044)
	Retired	31 (83.80)	6 (16.20)	0.12 (0.04–0.27, *p* < 0.001)	0.57 (0.18–1.52, *p* = 0.282)
	Working in governmental work	127 (39.00)	199 (61.00)	0.97 (0.75–1.27, *p* = 0.832)	1.24 (0.83–1.86, *p* = 0.295)
	Working in private sector	128 (48.30)	137 (51.70)	0.66 (0.50–0.88, *p* = 0.004)	1.17 (0.81–1.69, *p* = 0.403)
**Field of study or work**	Non-medical	593 (63.00)	349 (37.00)	-	-
	Medical	141 (20.50)	547 (79.50)	6.59 (5.26–8.30, *p* < 0.001)	4.30 (3.27–5.69, *p* < 0.001)
**Does the household income suffice the basic requirements of the family**	It barely suffices	386 (42.20)	528 (57.80)	-	-
	It is more than sufficient	100 (29.20)	242 (70.80)	1.77 (1.36–2.32, *p* < 0.001)	1.45 (1.06–2.00, *p* = 0.021)
	It is not sufficient	248 (66.30)	126 (33.70)	0.37 (0.29–0.48, *p* < 0.001)	0.54 (0.40–0.73, *p* < 0.001)
**Do you have health insurance**	No	448 (48.70)	471 (51.30)	-	-
	Yes	286 (40.20)	425 (59.80)	1.41 (1.16–1.72, *p* = 0.001)	1.12 (0.86–1.46, *p* = 0.406)
**Does the health insurance or monthly income support visiting a physician whenever you want**	No	493 (52.20)	451 (47.80)	-	-
	Yes	241 (35.10)	445 (64.90)	2.02 (1.65–2.47, p < 0.001)	1.11 (0.85–1.44, *p* = 0.463)
**History of drug allergy**	No	464 (45.20)	563 (54.80)	-	-
	Not sure	170 (43.90)	217 (56.10)	1.05 (0.83–1.33, *p* = 0.673)	1.08 (0.81–1.44, *p* = 0.582)
	Yes	100 (46.30)	116 (53.70)	0.96 (0.71–1.28, *p* = 0.765)	1.09 (0.76–1.57, *p* = 0.624)
**Medical history for chronic or congenital diseases**	No	466 (41.00)	670 (59.00)	-	-
	Yes	268 (54.30)	226 (45.70)	0.59 (0.47–0.73, *p* < 0.001)	1.02 (0.77–1.36, *p* = 0.875)

^*^ *P* – value significance ≤ 0.05

Abbreviations: *SD* Standard deviation, OR Odds ration, *aOR* adjusted odds ration, *CI* Confidence interval

### Univariate and multivariate analysis of the attitude

In the multivariate analysis of SM attitude, working or studying in the medical field was found to significantly increase the odds of practicing SM (aOR:1.96; CI: [1.52–2.53], *p*-value < 0.001) compared to the non-medical field. Having good knowledge significantly increases the odds of favorable attitudes (aOR:1.72; CI: [1.36–2.19], *p*-value < 0.001) (Table 7).

**Table 7:** Univariate and multivariate logistic regression analysis of attitude toward self-medication among the study participants

**Dependent: attitude level**		**Unfavorable**	**Favorable**	**OR (univariable)**	**aOR (multivariable)**
**Age**	Mean (SD)	32.4 (13.8)	31.0 (12.9)	0.99 (0.98–1.00, *p* = 0.031)	1.00 (0.99–1.02, *p* = 0.935)
**Gender**	Male	419 (52.9)	373 (47.1)	-	-
	Female	424 (50.6)	414 (49.4)	1.10 (0.90–1.33, *p* = 0.352)	1.05 (0.84–1.30, *p* = 0.682)
**Region or province**	Greater Cairo Region	210 (53.3)	184 (46.7)	-	-
	Alexandria Region	106 (50.2)	105 (49.8)	1.13 (0.81–1.58, *p* = 0.472)	1.17 (0.82–1.67, *p* = 0.379)
	Asyut Region	32 (52.5)	29 (47.5)	1.03 (0.60–1.78, *p* = 0.903)	0.75 (0.42–1.32, *p* = 0.312)
	Delta Region	155 (54.0)	132 (46.0)	0.97 (0.72–1.32, *p* = 0.855)	0.88 (0.63–1.23, *p* = 0.451)
	Northern Upper Egypt	65 (52.0)	60 (48.0)	1.05 (0.70–1.58, *p* = 0.800)	1.15 (0.75–1.76, *p* = 0.523)
	Southern Upper Egypt	215 (50.5)	211 (49.5)	1.12 (0.85–1.47, *p* = 0.418)	1.02 (0.76–1.37, *p* = 0.904)
	Suez Canal Region	60 (47.6)	66 (52.4)	1.26 (0.84–1.88, *p* = 0.267)	1.09 (0.71–1.66, *p* = 0.700)
**Residence**	Rural	342 (54.5)	285 (45.5)	-	-
	Urban	501 (50.0)	502 (50.0)	1.20 (0.98–1.47, *p* = 0.071)	1.11 (0.89–1.39, *p* = 0.354)
**Marital status**	Single	472 (49.6)	480 (50.4)	-	-
	Divorced	18 (64.3)	10 (35.7)	0.55 (0.24–1.17, *p* = 0.130)	0.78 (0.32–1.82, *p* = 0.570)
	Married	328 (53.3)	287 (46.7)	0.86 (0.70–1.05, *p* = 0.147)	1.34 (0.95–1.88, *p* = 0.092)
	Widow/Widower	25 (71.4)	10 (28.6)	0.39 (0.18–0.80, *p* = 0.014)	0.52 (0.20–1.24, *p* = 0.150)
**Highest educational degree**	High or secondary school	275 (53.2)	242 (46.8)	-	-
	Higher (university) education or above	516 (50.0)	517 (50.0)	1.14 (0.92–1.41, *p* = 0.229)	1.00 (0.78–1.28, *p* = 0.985)
	Primary or elementary	52 (65.0)	28 (35.0)	0.61 (0.37–0.99, *p* = 0.050)	0.87 (0.50–1.49, *p* = 0.612)
**Employment**	Not working	406 (50.6)	396 (49.4)	-	-
	Free work such as freelancer, dayworker	129 (64.5)	71 (35.5)	0.56 (0.41–0.78, *p* < 0.001)	0.78 (0.54–1.12, *p* = 0.186)
	Retired	15 (40.5)	22 (59.5)	1.50 (0.77–3.00, *p* = 0.233)	2.95 (1.34–6.71, *p* = 0.008)
	Working in governmental work	164 (50.3)	162 (49.7)	1.01 (0.78–1.31, *p* = 0.923)	0.95 (0.68–1.33, *p* = 0.768)
	Working in private sector	129 (48.7)	136 (51.3)	1.08 (0.82–1.43, *p* = 0.583)	1.26 (0.92–1.73, *p* = 0.153)
**Field of study or work**	Non-medical	565 (60.0)	377 (40.0)	-	-
	Medical	278 (40.4)	410 (59.6)	2.21 (1.81–2.70, *p* < 0.001)	1.96 (1.52–2.53, *p* < 0.001)
**Does the household income suffice the basic requirements of the family**	It barely suffices	468 (51.2)	446 (48.8)	-	-
	It is more than sufficient	157 (45.9)	185 (54.1)	1.24 (0.96–1.59, *p* = 0.095)	0.99 (0.76–1.30, *p* = 0.939)
	It is not sufficient	218 (58.3)	156 (41.7)	0.75 (0.59–0.96, *p* = 0.021)	1.06 (0.81–1.39, *p* = 0.669)
**Do you have health insurance**	No	493 (53.6)	426 (46.4)	-	-
	Yes	350 (49.2)	361 (50.8)	1.19 (0.98–1.45, *p* = 0.077)	0.96 (0.76–1.22, *p* = 0.756)
**Does the health insurance or monthly income support visiting a physician whenever you want**	No	526 (55.7)	418 (44.3)	-	-
	Yes	317 (46.2)	369 (53.8)	1.46 (1.20–1.79, *p* < 0.001)	1.28 (1.01–1.61, p = 0.041)
**History of drug allergy**	No	515 (50.1)	512 (49.9)	-	-
	Not sure	208 (53.7)	179 (46.3)	0.87 (0.68–1.09, *p* = 0.227)	0.99 (0.77–1.27, *p* = 0.930)
	Yes	120 (55.6)	96 (44.4)	0.80 (0.60–1.08, *p* = 0.149)	0.82 (0.60–1.12, *p* = 0.203)
**Medical history for chronic or congenital diseases**	No	576 (50.7)	560 (49.3)	-	-
	Yes	267 (54.0)	227 (46.0)	0.87 (0.71–1.08, *p* = 0.214)	1.00 (0.78–1.28, *p* = 0.995)
**Knowledge level**	Poor	454 (61.9)	280 (38.1)	-	-
	Good	389 (43.4)	507 (56.6)	2.11 (1.73–2.58, *p* < 0.001)	1.72 (1.36–2.19, *p* < 0.001)

^*^ *P* – value significance ≤ 0.05

Abbreviations: *SD* Standard deviation, *OR* Odds ration, *aOR* Adjusted odds ration, *CI* Confidence interval

### Univariate and multivariate analysis of the practice

In the multivariate analysis of SM practice, working or studying in the medical field was found to significantly increase the odds of practicing SM (aOR:1.65; CI: [1.26–2.17], *p*-value < 0.001) compared to the non-medical field. Having good knowledge slightly increases the odds of practicing SM, however, this was insignificant (aOR:1.15; CI: [0.90–1.48], *p*-value = 0.268). On the other hand, participants with favorable attitudes had significantly decreased odds of practicing SM (aOR:0.44; CI: [0.36–0.55], *p*-value < 0.001) (Table 8).

**Table 8:** Univariate and multivariate logistic regression analysis of self-medication practice among the study participants

**Dependent: Practice of SM**		**No**	**Yes**	**OR (95% CI, *p*-value) (univariable)**	**aOR (95% CI, *p*-value) (multivariable)**
**Age** (years)	Mean (SD)	31.0 (13.40)	32.2 (13.40)	1.01 (1.00–1.01, *p* = 0.065)	1.00 (0.99–1.02, *p* = 0.751)
**Gender**	Male	307 (38.80)	485 (61.20)	-	-
	Female	299 (35.70)	539 (64.30)	1.14 (0.93–1.40, *p* = 0.198)	1.24 (0.99–1.56, *p* = 0.065)
**Region or province**	Greater Cairo Region	147 (37.30)	247 (62.70)	-	-
	Alexandria Region	100 (47.40)	111 (52.60)	0.66 (0.47–0.93, *p* = 0.016)	0.66 (0.46–0.94, *p* = 0.023)
	Asyut Region	15 (24.60)	46 (75.40)	1.83 (1.01–3.49, *p* = 0.056)	1.70 (0.91–3.31, *p* = 0.107)
	Delta Region	83 (28.90)	204 (71.10)	1.46 (1.06–2.03, *p* = 0.023)	1.50 (1.06–2.14, *p* = 0.024)
	Northern Upper Egypt Region	52 (41.60)	73 (58.40)	0.84 (0.56–1.26, *p* = 0.390)	0.96 (0.62–1.49, *p* = 0.856)
	Southern Upper Egypt	158 (37.10)	268 (62.90)	1.01 (0.76–1.34, *p* = 0.948)	1.08 (0.80–1.47, *p* = 0.608)
	Suez Canal Region	51 (40.50)	75 (59.50)	0.88 (0.58–1.32, *p* = 0.524)	0.95 (0.62–1.46, *p* = 0.805)
**Residence**	Rural	232 (37.00)	395 (63.00)	-	-
	Urban	374 (37.30)	629 (62.70)	0.99 (0.80–1.21, *p* = 0.907)	1.08 (0.85–1.36, *p* = 0.528)
**Marital status**	Single	374 (39.30)	578 (60.70)	-	-
	Divorced	8 (28.60)	20 (71.40)	1.62 (0.73–3.94, *p* = 0.256)	1.38 (0.57–3.58, *p* = 0.488)
	Married	212 (34.50)	403 (65.50)	1.23 (1.00–1.52, *p* = 0.055)	1.24 (0.87–1.78, *p* = 0.233)
	Widow/Widower	12 (34.30)	23 (65.70)	1.24 (0.62–2.60, *p* = 0.552)	1.09 (0.46–2.71, *p* = 0.842)
**Highest educational degree**	High or secondary school	198 (38.30)	319 (61.70)	-	-
	Higher (university) education or above	383 (37.10)	650 (62.90)	1.05 (0.85–1.31, *p* = 0.640)	0.92 (0.72–1.18, *p* = 0.518)
	Primary or elementary education	25 (31.20)	55 (68.80)	1.37 (0.83–2.29, *p* = 0.227)	1.54 (0.88–2.76, *p* = 0.134)
**Employment**	Not working	325 (40.50)	477 (59.50)	-	-
	Free work such as freelancer, dayworker	70 (35.00)	130 (65.00)	1.27 (0.92–1.75, *p* = 0.153)	1.48 (1.03–2.16, *p* = 0.037)
	Retired	17 (45.90)	20 (54.10)	0.80 (0.41–1.57, *p* = 0.512)	0.84 (0.37–1.88, *p* = 0.662)
	Working in governmental work	104 (31.90)	222 (68.10)	1.45 (1.11–1.91, *p* = 0.007)	1.33 (0.94–1.90, *p* = 0.113)
	Working in private sector	90 (34.00)	175 (66.00)	1.32 (0.99–1.78, *p* = 0.058)	1.52 (1.09–2.12, *p* = 0.014)
**Field of study or work**	Non-medical	363 (38.50)	579 (61.50)	-	-
	Medical	243 (35.30)	445 (64.70)	1.15 (0.94–1.41, *p* = 0.185)	1.65 (1.26–2.17, *p* < 0.001)
**Does the household income suffice the basic requirements of the family** **Do you have health insurance**	It barely suffices	326 (35.70)	588 (64.30)	-	-
	It is more than sufficient	140 (40.90)	202 (59.10)	0.80 (0.62–1.03, *p* = 0.086)	0.80 (0.60–1.05, *p* = 0.111)
	It is not sufficient	140 (37.40)	234 (62.60)	0.93 (0.72–1.19, *p* = 0.549)	0.91 (0.69–1.20, *p* = 0.490)
	No	354 (38.50)	565 (61.50)	-	-
	Yes	252 (35.40)	459 (64.60)	1.14 (0.93–1.40, *p* = 0.202)	1.14 (0.90–1.45, *p* = 0.276)
**Does the health insurance or monthly income support visiting a physician whenever you want**	No	341 (36.10)	603 (63.90)	-	-
	Yes	265 (38.60)	421 (61.40)	0.90 (0.73–1.10, *p* = 0.301)	0.92 (0.72–1.17, *p* = 0.480)
**History of drug allergy**	No	405 (39.40)	622 (60.60)	-	-
	Not sure	134 (34.60)	253 (65.40)	1.23 (0.96–1.57, *p* = 0.097)	1.22 (0.94–1.59, *p* = 0.131)
	Yes	67 (31.00)	149 (69.00)	1.45 (1.06–1.99, *p* = 0.021)	1.35 (0.97–1.90, *p* = 0.074)
**Medical history for chronic or congenital diseases**	No	446 (39.30)	690 (60.70)	-	-
	Yes	160 (32.40)	334 (67.60)	1.35 (1.08–1.69, *p* = 0.008)	1.26 (0.98–1.63, *p* = 0.073)
**Knowledge level**	Poor	280 (38.10)	454 (61.90)	-	-
	Good	326 (36.40)	570 (63.60)	1.08 (0.88–1.32, *p* = 0.464)	1.15 (0.90–1.48, *p* = 0.268)
**Attitude level**	Unfavorable	245 (29.10)	598 (70.90)	-	-
	Favorable	361 (45.90)	426 (54.10)	0.48 (0.39–0.59, *p* < 0.001)	0.44 (0.36–0.55, *p* < 0.001)

^*^ *P* – value significance ≤ 0.05

Abbreviations: *SD* Standard deviation, *OR* Odds ration, *aOR* adjusted odds ration, *CI* Confidence interval

## Discussion

In the Arab world, SM is a widespread phenomenon that can seriously harm both the individual and the community [7]. To the authors’ knowledge, this is the largest study ever conducted on this wide scale including 1630 participants from all provinces of Egypt. We assessed the knowledge level of the population regarding the idea of SM, their attitudes, and practices regarding this phenomenon. We stated that most of the Egyptian individuals have good knowledge levels and unfavorable attitudes or perspectives regarding SM although most participants practice SM.

The present study shows a good average level of knowledge of the participants regarding SM, with around three-fourths of the participants aware of SM while only 62.80% practiced SM in the last three months. A recent multi-center study showed that Egypt had the highest prevalence of SM (72.10%) among the Arab countries during the COVID-19 pandemic [7]. Notably, most of our participants (58.30%) reported no effect of the COVID-19 pandemic on their habit of SM while only 12.00% reported an increase in SM during the pandemic. The participants’ economic status, which differed from the pandemic until recently, can contribute to the difference between SM practice rates in addition to the difference in participants’ characteristics.

Country-wise, despite having comparable rates of SM practice, only half of the Saudi Arabian population in the western region was aware of SM [25]. This discrepancy in awareness rates could be partially attributed to the cultural differences between country populations. It is noteworthy that half of our participants expressed unfavorable attitudes towards SM which could reflect cultural and behavioral backgrounds. Additionally, socio-economic status can be a contributing factor. Notably, more than half of our participants did not have health insurance or a monthly income that supports visiting the physician whenever they want.

Although two-thirds of our participants agreed that using medications without medical supervision can hide serious symptoms and may exaggerate conditions, painkillers were the most used by most participants followed by antibiotics used by one-third of participants. Analgesic and antibiotic misuse are well-established problems in Arab countries, especially Egypt. While analgesics can have numerous side effects, the rising challenge of antibiotic misuse results in serious effects such as antibiotic resistance, treatment failure, and even death in some cases [13, 26].

As the pharmacist’s recommendation was the primary source of SM in the present and previous multicenter studies [7], Egypt has no specific restrictions on dispensing analgesics and antibiotics from community pharmacies [13, 26]. Such problems are aggravated by the unnecessary and unfavorable practice of community pharmacists. While some of them may have enough knowledge to appropriately use antibiotics, most of them do not apply these regulations in clinical practice. The practice-knowledge gap was detected among community pharmacists and most of them dispensed antibiotics or symptomatic treatments without collecting the relevant information [13, 18].

In contrast to previous studies [7, 23, 27], our analysis shows no significant association between age and either the knowledge or practice of SM. Regarding gender, consistent with previous studies [20, 23, 25, 28], our study also shows that females had a significantly higher knowledge level about SM than males. This finding may be attributed to the biological nature of the woman’s body. Moreover, being more susceptible to certain health conditions than men (e.g., urinary tract infections and autoimmune diseases), besides the monthly menstrual cycle and its related pain, hormonal changes, and low immunity necessitates her to be aware of SM and certain drugs especially antibiotics and analgesics [29]. The cautious nature of females, in contrast to males, can also have an important contributing role [30]. Despite the high knowledge level among females, in our study, there was no significant association between gender and SM as it was in most previous studies [7, 23, 31, 32] females. This may indicate that practice is not only a reflection of knowledge level.

Participants in the medical field showed a significantly higher level of knowledge and favorable attitudes compared to participants in the non-medical field. This may be due to the nature of the medical occupations that make them more acquainted with the medications’ basic knowledge and awareness of SM than the general population. Moreover, this study shows participants belonging to a higher educational level scored higher knowledge levels than those of a lower educational level. Similar to the findings of the study in Mansoura city, working or studying in the medical field was substantially linked to increased probabilities of SM, which could be based on educational background and overconfidence [4].

While good knowledge was associated with favorable attitudes in the present study, there was no significant relationship between the knowledge level and practice of SM. This brings up the crucial fact that having good or relatively higher knowledge is not always translated into good practice. Favorable attitudes, however, were linked to a lower likelihood of engaging in SM. The populace may need to avoid overestimating the value of information since they may possess knowledge but lack the proper attitudes towards it or be affected by other variables that prevent them from making the best judgments. The potential effects and reflections that information may have on a population's attitudes, behaviors, and practices are what gives knowledge its power.

### Study strengths and limitations

To the authors’ knowledge, this is the largest study ever conducted on this wide scale of participants from all provinces of Egypt. Furthermore, the data was collected from several provinces which enhanced the generalizability of the study findings. We explored the pattern of and reasons for practicing SM among the Egyptians. To reduce the susceptibility of recall bias, we specified the duration of practicing SM to only the last three months rather than asking if ever practiced SM. Due to the lack of extensively updated data about SM in Egypt, this study can help in redefining some regulations and policies, especially regarding dispensing medications.

On the other side, our study does have some limitations. The used questionnaire was self-administered which can cause information bias in our results as some people may not understand questions properly. Besides, it was susceptible to social desirability bias as some participants may tend to give favorable answers. The Cronbach’s alpha score for the knowledge section was 0.62 which can be considered below the acceptable level of reliability for some references. Our data may not have had an appropriate representation of all age groups due to the difference in using social media for the younger although our collaborators tried to avoid this by distributing the paper survey among the older age groups.

### Recommendations and future implications

The main sources of SM were older medications at home or from outdated prescriptions. Therefore, it is necessary to educate the public about such negative habits and their potential negative effects. Healthcare professionals ought to always instruct the general public on this information, not just when to use those medications. Our efforts should focus on not only imparting sound knowledge but also on figuring out how to translate that level of understanding into positive behaviors. The behaviors and practices of community pharmacists also have a role and thus, public education about the pharmacists’ actual job is needed in addition to certain regulations on dispensing medication without a physician's approval. Providing quality medical services and improving the accessibility of medical services and the coverage of health insurance is also needed.

Concerning future research, more thorough studies on the population with more focus on non-medical individuals and representative samples of the age groups and other demographics are needed. Investigating the underlying reasons for such practice on the national level can help tailor the required interventions. Consequently, proper restrictions will need to be applied by the national healthcare authorities. If the phenomenon of SM increases significantly worldwide, international guidelines for dispensing and using medications might be considered for changes.

## Conclusions

SM is considered a part of self-care when it follows the regulations and guidelines. This study indicates that the Egyptian population has a high prevalence of SM despite the high knowledge level and good or favorable attitude toward SM. Many factors are contributing to this phenomenon. However, the cultural point of view plays an important role. This rising issue should be tackled by health institutions and governments to prevent individual and community health hazards.

### Supplementary Information

**Additional file 1.**

## Data Availability

Data is available upon the reasonable request of the corresponding author.

## Abbreviations

SM: Self-medication
OTCs: Over-the-counter drugs
POMs: Prescription-only medications
CDC: The Centre for Disease Control and Prevention
WHO: The World Health Organization
COVID-19: Coronavirus disease 2019
KAP: Knowledge, attitude, and practice
CI: Confidence interval
OR: Odds ratio
aOR: Adjusted odds ratio
