# Supplementary material for: Unravelling the dilemma of self-medication in Egypt: a cross-sectional survey on knowledge, attitude, and practice of the general Egyptian population
Source: BMC Public Health. 2024 Mar 1;24:652. doi: 10.1186/s12889-024-17913-3 (PMC10905903; doi:10.1186/s12889-024-17913-3)
Supplement: Supplementary file 1 — Additional file 1. [file 12889_2024_17913_MOESM1_ESM.pdf]

Unravelling the Dilemma of Self-Medication in Egypt: A Cross-Sectional Survey on Knowledge, Attitude, and Practice of the General Egyptian Population

Hossam Tharwat Ali [1,], Mostafa Barakat [2], Ahmed Reda Abdelhalim [3], Ibrahim Noureddin Al-Kurd [4], Muhammad Kamal-Eldeen Muhammad [5], Mohamed Mostafa Sharkawy [6], Mohamed Elbahnasawy [7], Samar Ahmed Amer [8], and Self-Medication Collaborative Group*

English Version of The Study Questionnaire

Informed Consent

This is a cross-sectional study to assess the knowledge, prevalence, patterns, and practices of self-medication without the supervision or prescription of a specialized physician among adults in Egypt, as well as their attitudes towards this phenomenon. The study aims to understand these aspects for developing future plans to face this phenomenon and raise awareness among the general public. The study will not use any personal or identifiable information with the participants.

Do you agree to participate in this study?

- Yes ***(Proceed to the questionnaire)***
- No

Have you participated in this study or filled out this questionnaire before?

- Yes ***(Not included to prevent duplicate data)***
- No ***(Proceed to the questionnaire)***

Demographic characteristics

1- Age in years: ……….

2- Gender:

- Male
- Female

3- Region or province

- Cairo Region (Cairo / Giza / Qalyubia)
- Alexandria Region (Alexandria / Beheira / Matrouh)
- Delta Region (Gharbia / Dakahlia / Kafr El Sheikh / Menoufia / Damietta)
- Suez Canal Region (Ismailia / Suez / Port Said / North Sinai / South Sinai)
- Northern Upper Egypt Region (Fayoum / Minya / Beni Suef)

- Central Upper Egypt Region (Assiut / New Valley)
- Southern Upper Egypt Region (Qena / Sohag / Luxor / Aswan / Red Sea)

4- Residence:

- Urban
- Rural

5- Marital status

- Single
- Married
- Widow/Widower
- Divorced

6- Highest educational degree

- Higher (university) education or above
- High or secondary school
- Primary or elementary education

7- Employment

- Not working
- Working in governmental work
- Working in private sector
- Free work such as freelancer, dayworker
- Retired

8- Field of study or work

- Non-medical
- Medical (Medicine / Pharmacy / Physical Therapy / Dentistry / Veterinary Medicine / Nursing)

9- Does the household income suffice the basic requirements of the family?

- It barely suffices
- It is not sufficient
- It is more than sufficient

10- Do you have health insurance?

- No
- Yes

11- Does the health insurance or monthly income support visiting a physician whenever you want?

- No
- Yes

12- History of drug allergy

- No
- Not sure
- Yes

13- History of chronic or congenital diseases?

- Cardiovascular diseases e.g. hypertension

- Musculoskeletal disorders
- Endocrinal disorders e.g. diabetes mellitus
- Chronic headache
- Respiratory conditions e.g. asthma
- Blood diseases e.g. anemia
- ENT disorders e.g. sinusitis
- Kidney diseases
- Liver diseases
- GIT condition
- Others: ………………………..

Knowledge regarding self-medication:

Please answer the following questions with one of the choices (Yes – No – Not sure). The correct answer is written beside each question.

1- **Self-medication is taking medications without prescription or supervision of specialized physician. Yes**
2- **Taking medications of unknown sources or origin such as herbals is always safe. No**
3- **In case side effects occur, we should consult a physician right away. Yes**
4- **Increasing the doses of medications without physician's supervision is always safe. No**
5- **Taking medication without physician's supervision can hide serious symptoms or conditions and lead to its exaggeration. Yes**

Attitude towards self-medication

Please answer the following questions with one of the choices:
(Completely disagree – Disagree - Uncertain / No difference – Agree - Strongly agree).

1- **Self-medication is a part of self-care.**
2- **The general population can prescribe medications properly without medical training.**
3- **Some people can properly recognize and diagnose diseases without consulting a physician.**
4- **Some people can properly take medications on their own without consulting a physician.**
5- **Self-medication is safe and I recommend it to my people.**

<u>Practice of self-medication</u>

1- Have you taken medications on your own without the physician’s supervision or prescription during the last three months?

- Yes
- No

<u>Participants who answered yes will complete the rest of the practice questions.</u>

2- How many times have you taken those medications in the last three months?

- Less than 3 times
- 3 - 6 times
- More than 6 times

3- Do these medications help you feel better?

- It gets worse.
- No difference / I rarely feel better.
- Sometimes I feel better.
- I always feel better

4- Where did you obtain those medications without a human doctor's prescription?
<u>*(Up to three choices) *</u>

- From friends
- From family or relatives
- From a pharmacist's recommendation
- Reusing old prescriptions
- From old medications at home
- Other: [Specify]

5- What medications have you obtained self-medication in the past 3 months?
<u>*(Up to three choices) *</u>

- Allergy medications
- Acne or skin problem medications
- Fever reducers
- Vitamins and dietary supplements
- Sleep aids
- Steroids (cortisone)
- Birth control
- Antibiotics
- Herbals
- Eye drops/ointments

- Medications for heartburn
- Cough medications
- Laxatives
- Pain relievers
- Medications for digestive problems
- Other: [Specify]

6- Which of the following did you know before taking those medications?
***(Up to three choices) ***

- Indications for use
- Medication name
- How to use or take the medication
- Proper dosage
- Possible side effects
- Duration of use or intake
- Contraindications
- Proper storage methods at home

7- Why did you take medications without consulting a healthcare professional?
***(Up to three choices) ***

- More privacy than going to the doctor
- Lack of sufficient time to visit a doctor
- Needed a quick response
- Lack of nearby hospitals or clinics
- Sufficient knowledge about my health issues from past experiences
- Mild or minor symptoms
- Embarrassment of visiting and examining a doctor
- Fear of hospital or clinic infections
- Financial constraints or lack of health insurance to visit a doctor
- Other: [Specify]

8- How did the COVID pandemic affect your habit of self-medication?

- Increased since the COVID-19 pandemic
- No difference (The COVID-19 pandemic did not affect this habit)
- Decreased (or stopped) since the COVID-19 pandemic
- I don't remember

9- Did you have any side effects that necessitate physician consultation?

- Yes
- No
- Not sure
